# The Turkish Neonatal Jaundice Online Registry: A national root cause analysis

**DOI:** 10.1371/journal.pone.0193108

**Published:** 2018-02-23

**Authors:** Omer Erdeve, Emel Okulu, Ozgur Olukman, Dilek Ulubas, Gokhan Buyukkale, Fatma Narter, Gaffari Tunc, Begum Atasay, Nazli Dilay Gultekin, Saadet Arsan, Esin Koc, on behalf of the Turkish Neonatal Jaundice Registry Collabolators

**Affiliations:** 1 Department of Pediatrics, Division of Neonatology, Ankara University School of Medicine, Ankara, Turkey; 2 Department of Pediatrics, Division of Neonatology, Behcet Uz Children’s Hospital, Izmir, Turkey; 3 Department of Neonatology, Etlik Zubeyde Hanım Women’s Health Teaching and Research Hospital, Ankara, Turkey; 4 Department of Pediatrics, Division of Neonatology, Kanuni Sultan Suleyman Training and Research Hospital, Istanbul, Turkey; 5 Department of Neonatology, Kartal Lutfi Kirdar Education and Training Hospital, Istanbul, Turkey; 6 Department of Pediatrics, Division of Neonatology, Necmettin Erbakan University, Meram School of Medicine, Konya, Turkey; 7 Department of Pediatrics, Division of Neonatology, Gazi University School of Medicine, Ankara, Turkey; TNO, NETHERLANDS

## Abstract

**Background:**

Neonatal jaundice (NNJ) is common, but few root cause analyses based on national quality registries have been performed. An online registry was established to estimate the incidence of NNJ in Turkey and to facilitate a root cause analysis of NNJ and its complications.

**Methods:**

A multicenter prospective study was conducted on otherwise healthy newborns born at ≥35 weeks of gestation and hospitalized for only NNJ in 50 collaborator neonatal intensive care units across Turkey over a 1-year period. Patients were analyzed for their demographic and clinical characteristics, treatment options, and complications.

**Results:**

Of the 5,620 patients enrolled, 361 (6.4%) had a bilirubin level ≥25 mg/dL on admission and 13 (0.23%) developed acute bilirubin encephalopathy. The leading cause of hospital admission was hemolytic jaundice, followed by dehydration related to a lack of proper feeding. Although all infants received phototherapy, 302 infants (5.4%) received intravenous immunoglobulin in addition to phototherapy and 132 (2.3%) required exchange transfusion. The infants who received exchange transfusion were more likely to experience hemolytic causes (60.6% vs. 28.1%) and a longer duration of phototherapy (58.5 ± 31.7 vs. 29.4 ± 18.8 h) compared to infants who were not transfused (p < 0.001). The incidence of short-term complications among discharged patients during follow-up was 8.5%; rehospitalization was the most frequent (58%), followed by jaundice for more than 2 weeks (39%), neurological abnormality (0.35%), and hearing loss (0.2%).

**Conclusions:**

Severe NNJ and bilirubin encephalopathy are still problems in Turkey. Means of identifying at-risk newborns before discharge during routine postnatal care, such as bilirubin monitoring, blood group analysis, and lactation consultations, would reduce the frequency of short- and long-term complications of severe NNJ.

## Introduction

Neonatal jaundice (NNJ) is common in the neonatal period due to the adaptation of bilirubin metabolism that occurs at this time. The majority of newborns develop NNJ, which is the most common cause of hospital admission or rehospitalization in the first week of life [1]. The advent of maternal rhesus immunoglobulin prophylaxis, phototherapy, and exchange transfusion reduced the rates of bilirubin-induced mortality and morbidity. However, acute bilirubin encephalopathy and kernicterus are still reported in low- and middle-income countries (LMICs) and even high-income countries (HICs) [1–6].

Despite its frequency, few root cause analyses of NNJ using national registries have been performed [1–6]. The incidence of NNJ in developed countries is reportedly 25–41, 17–45, and 2–36 cases per 100,000 live births for serum bilirubin levels above the exchange transfusion threshold, >25 mg/dL, and 30 mg/dL, respectively [5–7]. However, studies in LMICs are generally hospital based [2,3].

NNJ is the leading cause of admission to neonatal intensive care units (NICUs) among otherwise healthy newborns in Turkey, but there are no national data on NNJ and related complications in Turkey. Therefore, the Turkish Neonatal Society established a prospective online registry to estimate the incidence of severe NNJ and facilitate a root cause analysis of NNJ and its complications. The results will enable the identification of risk reduction strategies.

## Materials and methods

After the Turkish Neonatal Jaundice Online Registry was established in September 2015, a multicenter prospective observational cohort study was conducted among otherwise healthy infants born at ≥35 weeks of gestation and hospitalized for only NNJ. Clinical directors in NICUs nationwide were made aware of the study, and 50 NICUs participated for the 1-year study period. The NICUs were asked to add to the registry database daily all hospitalized cases of NNJ at discharge using an online, standard, patient specific electronic case report form (eCRF). The study was approved by the Online Studies Scientific Steering Committee of the Turkish Neonatal Society and by the Ankara University institutional review board. Written informed consent was obtained from the parents or guardians of the newborns.

Otherwise healthy newborns with NNJ referred to the hospital by maternity services, emergency services, and policlinics were enrolled in the study. Infants born at<35 weeks of gestation or with a diagnosis at admission unrelated to hyperbilirubinemia were excluded. The eCRF included demographic, clinical, and laboratory findings on the infants. Postnatal age, birth weight, gestational age, sex, delivery type, history of siblings with jaundice, weight loss on admission, total bilirubin level (mg/dL), bilirubin/albumin ratio, reticulocyte count, direct Coombs test positivity on admission, defined cause of hyperbilirubinemia, and treatments provided to the infants (phototherapy, exchange transfusion, intravenous immunoglobulin [IVIG]) were recorded.

Patients were managed in accordance with the unit protocols recommended by the American Academy of Pediatrics (AAP) for the management of hyperbilirubinemia in infants born at ≥35 weeks of gestation, which were commonly used by NICUs in Turkey during the study period [8]. The type and duration of phototherapy were recorded. A bilirubin level ≥25 mg/dL was considered to indicate severe NNJ, as an infant with jaundice of this magnitude is thought to be at high risk for kernicterus [9]. Hemolytic disorders were defined as the presence of anemia; higher than normal for postnatal age reticulocyte counts; a peripheral smear suggestive of hemolysis; and corroborating signs, such as direct Coombs test positivity. IVIG was used for exchange transfusion in patients with a borderline bilirubin level in hemolytic cases [10]. Exchange transfusion was performed according to the AAP criteria [8]. The bilirubin-induced neurological dysfunction score was used to identify acute bilirubin encephalopathy [7]. Patients’ follow-up findings for 3 months after discharge, including results of a physical examination, hearing test, and cerebral imaging, were recorded. All patients discharged from NICUs must undergo auditory evaluations to determine the auditory brainstem response at the time of discharge and at 2 to 3 months of age, according to the National Hearing Screening Program of Turkey. Abnormal neurological findings, such as decreased muscle tone, irritability, poor sucking, and seizures, were recorded at discharge and during follow-up. Severe cases of NNJ were followed up by neurologists, and cerebral imaging was performed at their discretion. Readmission was defined as a return to the hospital within 0–20 days of initial discharge [9].

Electronic data were summarized at the end of the study using descriptive statistics. Categorical data are presented as frequencies and percentages. We calculated means and SDs for continuous variables using Student’s t test. Fisher’s exact test was used to perform statistical comparisons. The chi-square test was used to test associations between categorical variables. p < 0.05 was taken to indicate statistical significance.

## Results

From September 2015 to September 2016, 5,620 hospitalized cases of NNJ were recorded. Only 2% of the patients (n = 112) were born at home. Characteristics of the patients are shown in Table 1. The patients’ median age at admission was 3 days, and their mean peak bilirubin level was 17.6 ± 4.8 mg/dL. At admission, 361 patients (6.4%) had a bilirubin level higher than the threshold for severe NNJ.

**Table 1:** Baseline demographic characteristics.

Characteristics	No. of infants (N = 5,620)
**Gestational age, weeks, mean (SD)**	37.9 (1.6)
**Sex (male), n (%)**	3,052 (53.3)
**Birth weight, g, mean (SD)**	3,102 (517)
**Mode of delivery (C/S), n (%)**	2,995 (53.3)
**History of siblings with jaundice, n (%)**	669 (11.9)
**Weight loss at presentation ≥10%, n (%)**	698 (12.4)
**Weight loss at presentation ≥15%, n (%)**	124 (2.2)

C/S cesarean section.

Table 2 shows the causes of admissions. The leading cause of hospital admissions was hemolytic jaundice, followed by dehydration related to a lack of proper feeding. No underlying condition was reported in 18.4% of the cases. ABO blood incompatibility was the most common cause of hemolytic jaundice (73.7%). The rate of rhesus incompatibility was similar in patients born at home and in the hospital (5.3 vs. 5.4%, respectively; p > 0.05). The frequencies of direct Coombs positivity, reticulocytosis (>7%), and a bilirubin/albumin ratio >6.5 were 13.1%, 6.7%, and 10.5%, respectively.

**Table 2:** Underlying conditions of infants with hyperbilirubinemia.

Etiology	No. of infants (N = 5,620)
**Hemolytic jaundice, n (%)**	1,624 (28.9)
ABO incompatibility	1,197 (21.3)
Rhesus incompatibility	304 (5.4)
Subgroup incompatibility	47 (0.8)
G6PD deficiency	28 (0.5)
Other	48 (0.9)
**Dehydration due to lack of proper feeding, n (%)**	1,551 (27.6)
**Breast milk jaundice, n (%)**	708 (12.6)
**Prematurity, n (%)**	478 (8.5)
**Cephalohematoma, n (%)**	123 (2.2)
**Polycythemia, n (%)**	102 (1.8)
**Unknown, n (%)**	1,034 (18.4)

G6PD, glucose 6-phosphate dehydrogenase.

Phototherapy of a mean duration of 30 ± 19 h was given to all patients, and the mean bilirubin level at the end of treatment was 10.3 ± 2.3 mg/dL. The majority of patients (82.6%) received phototherapy with light-emitting diode (LED), whereas 17.8% received conventional phototherapy. The rate of rehospitalization was not significantly higher among infants who received conventional phototherapy than those who received LED phototherapy (11.9% vs. 7.9, respectively; p > 0.05). Moreover, 302 infants (5.4%) received IVIG in addition to phototherapy. Exchange transfusion was performed in 132 patients (2.3%), including 39 infants who also received IVIG. Finally, 65 among 361 severe NNJ patients (18%) required exchange transfusion during their hospitalization.

Infants who received exchange transfusion were more likely to experience a hemolytic cause (60.6% vs. 28.1%) and a longer duration of phototherapy (58.5 ± 31.7 vs. 29.4 ± 18.8 h) than infants who were not transfused (p < 0.001). A multiple logistic regression model demonstrated that direct Coombs positivity (OR 2.6, 95% CI 1.45–4.8, p = 0.001), reticulocyte count ≥7% (OR 7.1, 95% CI 3.7–13.4, p = 0.001), and a bilirubin/albumin ratio ≥6.5 (OR 19.8, 95% CI 11.8–33.4, p = 0.001) were independently associated with an increased risk for exchange transfusion in infants with hyperbilirubinemia.

The 13 patients with acute bilirubin encephalopathy constituted 0.23% of the total cohort but 3.6% of the infants with severe NNJ. Table 3 summarizes the clinical, demographic, and laboratory findings for infants with ABE. The mean gestational age and birth weight of these infants were 38.3 ± 1.5 weeks and 3,060 ± 640 g, respectively. Infants with features that suggested encephalopathy were more likely to be male (11/13). They had significantly higher peak serum bilirubin levels (mean 35.9 ± 5.7 mg/dL), and all of them underwent exchange transfusion. Two patients died despite all treatments. A multiple logistic regression model demonstrated that male sex, a hemolytic cause, reticulocytosis, and a high bilirubin/albumin ratio were associated with an increased risk for ABE in infants (p < 0.05).

**Table 3:** Characteristics of patients with acute bilirubin encephalopathy.

No.	GA (week)	BW (g)	Sex	Mode of delivery	Age on Admission (d)	TSB on Admission (mg/dL)	Underlying Condition	Complication on Follow-up
**1**	38	3,600	F	C/S	5	35,0	Hemolytic jaundice	Neurological abnormality
**2**	40	3,650	M	C/S	5	43,0	Unknown	Neurological abnormality
**3**	36	2,200	M	NVD	6	28,6	Hemolytic jaundice	Neurological abnormality
**4**	38	3,200	M	NVD	5	36,0	Hemolytic jaundice	Neurological abnormality
**5**	39	2,200	M	NVD	4	32,0	Hemolytic jaundice	Neurological abnormality
**6**	38	4,000	M	NVD	2	29,0	Hemolytic jaundice	Hearing loss
**7**	38	3,200	M	C/S	4	32,0	Unknown	Prolonged jaundice
**8**	41	2,670	F	NVD	3	38,6	Lack of proper feeding	Neurological abnormality
**9**	37	3,150	M	NVD	5	33,0	Hemolytic jaundice	Neurological abnormality
**10**	40	4,000	M	NVD	2	44,0	Hemolytic jaundice	Hearing loss
**11**	38	2,300	M	NVD	4	30,2	Unknown	Prolonged jaundice
**12**	39	3,050	M	NVD	3	42,0	Hemolytic jaundice	Died from sepsis
**13**	36	2,560	M	NVD	4	43,1	Lack of proper feeding	Died from multiorgan dysfunction

GA, gestational age; BW, birth weight; C/S, cesarean section; NVD, normal vaginal delivery; TSB, total serum bilirubin.

At follow-up, the incidence of short-term complications among discharged patients was 8.5% (n = 478), and rehospitalization (58%) was the most common complication. Other complications were prolonged jaundice (39%), neurological abnormality (0.35%), and hearing loss (0.2%). The major etiologies in readmitted infants were hyperbilirubinemia due to hemolytic causes (46%) and dehydration related to poor feeding (26%).

## Discussion

A detailed root cause analysis would enable the identification of strategies to improve the efficacy of NNJ treatment [3,6]. We conducted a nationwide root cause analysis of NNJ that, to the best of our knowledge, involved the largest population of any similar study and also evaluated treatment options and postdischarge follow-up data. The findings presented here are generally consistent with the literature, although there are several noteworthy observations. First, severe NNJ and ABE continue to occur in Turkey at frequencies higher than those in HICs but lower than those in LMICs [2,3]. The incidence of severe NNJ and bilirubin encephalopathy among NNJ patients was 6.4% and 2.3%, respectively. Second, the two most common risk factors for NNJ were a hemolytic etiology and lack of initiation of proper breastfeeding. Third, the rate of cesarean section (C/S) was higher than that recommended by the World Health Organization (WHO), and it might lead to a delay in establishment of proper breastfeeding [11]. Fourth, in spite of Rhesus anti-D prophylaxis being provided free by the state, Rhesus immunization is still the second most common cause of hemolytic jaundice.

The majority of national studies involve voluntary reporting of severe NNJ or kernicterus cases [5–7]. A study of Swedish birth registry data that involved a large, nationwide population indicated that maternal risk factors, such as older age, blood type, high body mass index, and parity, were associated with an increased risk for NNJ. However, this study was limited, as bilirubin levels were not determined and infant characteristics associated with NNJ were not assessed [12]. The introduction of the Canadian Pediatric Society guidelines and improved physician awareness of severe NNJ in the past 10 years have likely made positive contributions, but this analysis was a cross-sectional comparison involving a small population [9]. A large retrospective cohort study in England evaluated term admissions to all NICUs and found that NNJ was the second leading cause of admissions overall and the leading cause of admissions from home. Although this study included outcomes related to the effects of transitional care on NNJ, it was not a root cause analysis and did not include postdischarge follow-up data [13]. For practical reasons, studies of the effectiveness of strategies to reduce the incidence of severe NNJ and ABE rely on surrogate acute outcomes, such as serum bilirubin level, readmission for phototherapy, use of exchange transfusion, and jaundice-associated neonatal mortality [14].

The national health system should recognize severe NNJ as a priority and invest resources in addressing this tragic but largely preventable condition. Safe and effective care of both healthy and sick infants requires a seamless transition from a birthing facility to home following discharge. The unacceptable occurrence of severe NNJ and ABE or kernicterus is due to deficiencies in the healthcare system as well as inadequate follow-up during the first week of life [15]. Since the length of hospital stay for newborns was reduced in the early 1990s, the frequency of neonatal readmission to hospital has increased [13,16]. Our results raise concerns that underrecognition of NNJ in otherwise healthy infants leads to early readmission and may have long-term consequences.

Turkish Neonatal Jaundice Registry data demonstrated that hemolytic jaundice was the major cause of NNJ, followed by a lack of proper feeding. ABO incompatibility was the most common cause of hemolytic jaundice, followed by Rh disease (18.7%), and was higher than reported rates in HICs. The frequency of hyperbilirubinemia of undetermined etiology in our study was 18.4%, which is consistent with previous reports (17% to 37%) [3,17,18].

The etiology of severe NNJ differs depending on genetic factors and geographical location, even within regions of the same country [3]. The Child Health Epidemiology Reference Group (CHERG) modeling study estimates that 78% of cases of extreme hyperbilirubinemia are attributable to Rh disease, 6% to glucose 6-phosphate dehydrogenase deficiency (G6PD), 2% to moderate/late preterm birth, and 15% to other causes [19]. Moreover, 80% of affected infants are in countries with a mortality rate of >15/1,000 live births. The model assumes no severe NNJ attributable to Rh disease in HICs because of effective prevention and treatment. By contrast, it addresses the high cost of immune prophylaxis in LMICs [19]. In a study of pregnant women in Turkey, the most common ABO blood group was O positive (38.7%), and 15.4% of subjects were Rh negative. Indeed, the prevalence of alloimmunization was 8.7% in the Rh-negative group. Despite prophylaxis, rhesus disease remains a common cause of alloimmunization in Turkey [20]. All pregnant women and infants in Turkey are covered by the state insurance system, but the lack of routine maternal and neonatal blood testing increases the frequency of ABO incompatibility, which is a risk factor for severe NNJ. There is also evidence that NNJ due to Rh disease is a more important cause of brain damage than other forms of hyperbilirubinemia, as observed in our bilirubin-induced encephalopathy series. Thus, blood type screening and effective prophylaxis should be provided to pregnant women in Turkey.

The frequency of G6PD deficiency in Turkey varies according to geographic location and ethnicity. A previous study in a referral center reported that of 4,906 patients hospitalized for hyperbilirubinemia, 55 were G6PD deficient [21]. The frequency of G6PD deficiency in this study was lower than that reported previously and the CHERG modeling estimates [19,21].

Two striking findings of this study are the high rates of C/S in Turkey and NNJ due to a lack of proper breastfeeding. The incidence of C/S is increasing worldwide; this may be because of the decrease in the rate of vaginal delivery after a previous C/S, the increase in the frequency of high-risk pregnancies, the delayed age at marriage, and the increase in requests for C/S [22,23]. The appropriate length of hospital stay following C/S is debated. The Turkish Ministry of Health recommends discharge at 3 days after an uncomplicated C/S. Indeed, early discharge reportedly decreases both the rate of iatrogenic infection and hospital costs, and mothers are commonly discharged on the second day. However, early discharge impedes breastfeeding because milk flow may not be established until days 3 or 4 postpartum, and a short stay is insufficient for a new mother to learn about breastfeeding [24]. A systematic review and meta-analysis showed that C/S reduced the rate of breastfeeding compared to vaginal birth [22]. Bayoumi *et al*. compared maternal and neonatal outcomes of patients discharged 24 versus 72 h after C/S; the neonatal admission rate was significantly higher in the 24 h group, mainly because of NNJ [25]. Erdeve *et al*. reported that C/S increased the risk for hypernatremic dehydration 2.4-fold compared to vaginal delivery [26]. Unsuccessful breastfeeding due to C/S delivery and insufficient lactation consultation might lead to a lack of proper feeding, resulting in NNJ. Estimates of the incidence of severe NNJ in developed countries are 2 to 12/100,000, with the lowest rates in Switzerland and the United States [27–32]. Such lower levels could be due to established care practices, such as the implementation of universal screening in US hospitals and longer postpartum stays following vaginal delivery in Switzerland [30,32].

Acute and chronic bilirubin encephalopathy are largely preventable if severe NNJ is identified early and treated promptly with phototherapy or exchange transfusion. Mreihil *et al*. reported that the rate of kernicterus in Norway is 1:600,000, and this single case occurred because national guidelines for follow-up were not followed [33]. By contrast, a lack of effective phototherapy units, particularly those capable of providing intensive irradiance, is associated with severe NNJ [10,34]. Moreover, a recent study from Norway demonstrated that despite the existence of national uniform guidelines for phototherapy, phototherapy practices, particularly those related to irradiance and distance, varied among NICUs [35]. The majority of patients in the NICUs in our study received phototherapy with LEDs. The rate of rehospitalization among infants who received conventional phototherapy was not significantly higher, possibly because of differences in phototherapy practices among the NICUs. However, we did not record the irradiance and distance values used in phototherapy. Mreihil *et al*. reported that more than 25% of all infants admitted to Norwegian NICUs receive phototherapy. Of these infants, only 19.8% had a jaundice-related diagnosis as the principal reason for NICU admission, and the rate of phototherapy was inversely related to gestational age and birthweight [35]. The present study enrolled only patients with NNJ as the principal diagnosis; therefore, the importance of severe NNJ in preterm infants <35 weeks should be emphasized given its toxicity in the smallest, most vulnerable infants. The requirement for ET in developed countries has declined, largely because of improved surveillance of infants with clinically significant jaundice, routine use of rhesus immunoglobulin prophylaxis to prevent primary isoimmunization of Rh-negative women, and optimization of blue-light phototherapy. ET was performed in 132 of the infants (2.3%) in our study. The most common cause of ET was a hemolytic condition, similar to previous reports [36,37].

Population-based studies in the United States and Europe suggest that kernicterus occurs in about 0.5–1.0 per 100.000 infants born after 35 weeks of gestation, and the incidence of bilirubin encephalopathy in Canada is reportedly1 in 44,000–67,000 live births. The clinical risk factors associated with neurotoxicity at lower serum bilirubin levels include hemolytic disease, G6PD deficiency, prematurity, asphyxia, acidosis, sepsis, and hypoalbuminemia [2,3,6,38]. The incidence of ABE in our cohort was 0.23%, and the risk was associated with male sex, hemolytic causes, reticulocytosis, and a high bilirubin/albumin ratio.

It is often difficult for family practitioners or other primary care providers to evaluate all infants within 48–72 h of discharge, as recommended by the AAP [8]. Bhutani and colleagues reported that the serum bilirubin level of infants prior to hospital discharge is predictive of NNJ [39]. Bilirubin testing in all patients at discharge would allow for follow-up planning and further testing to evaluate the risk for and prevent severe NNJ. This would reduce the rate of hospitalization, severe NNJ, and exchange transfusion in addition to bilirubin-induced encephalopathy and mortality.

To the best of our knowledge, this is the first prospective, multicenter, online nationwide study. The main strength of this study is the large data set, which included NNJ admissions to 50 NICUs across Turkey. Therefore, the findings can be generalized to the national level. A further strength is the high level of eCRF completeness required, which resulted in the provision of all required information. The major limitation of the study is its reliance on self-reported data. Voluntary reporting leads to underreporting; therefore, the incidence of severe NNJ and bilirubin encephalopathy was likely underestimated. However, the data in the registry database were largely from tertiary hospitals with attending neonatologists who are aware of the condition.

Our results demonstrate that severe NNJ and bilirubin encephalopathy remain problems in Turkey. Despite advances in neonatal care, NNJ still results in severe morbidity. A considerable number of infants were readmitted soon after having been discharged from maternity services. Means of identifying at-risk newborns before discharge during routine postnatal care, such as bilirubin monitoring, blood group analysis, and lactation consultations, would reduce the frequency of short- and long-term complications of severe NNJ.

## Supporting information

S1 File Patient data. (XLSX)

## References

[1] Sgro M, Campbell D, Shah V. Incidence and causes of severe neonatal hyperbilirubinemia in Canada. CMAJ. 2006;175: 587–90. doi: 10.1503/cmaj.060328 16966660 10.1503/cmaj.060328 PMC1559442

[2] Olusanya BO, Ogunlesi TA, Kumar P, Boo NY, Iskander IF, de Almedia MF, et al Management of late-preterm and term infants with hyperbilirubinaemia in resource-constrained settings. BMJ Pediatr. 2015;15: 39. 10.1186/s12887-015-0358-z PMC4409776 25884679

[3] Greco C, Arnolde G, Boo NY, Iskander IF, Okolo AA, Rohsiswatmo R, et al Neonatal jaundice in low- and middle-income countries: Lessons and future directions from the 2015 Don Ostrow Trieste Yellow Retreat. Neonatology. 2016;110: 172–80. doi: 10.1159/000445708 27172942 10.1159/000445708

[4] Bratlid D, Nakstad B. Hansen TWR. National guidelines for treatment of jaundice in the newborn. Acta Paediatr. 2011;100: 499–505. doi: 10.1111/j.1651-2227.2010.02104.x 21114525 10.1111/j.1651-2227.2010.02104.x

[5] Pratesi S, Dani Carlo, Raimondi F, Romagnoli C. The Italian registry of kernicterus and hyperbilirubinemia. J Matern Fetal Neonatal Med. 2012;25: 118–20. doi: 10.3109/14767058.2012.714998 22958040 10.3109/14767058.2012.714998

[6] Kaplan M, Bromiker R, Hammerman C. Severe neonatal hyperbilirubinemia and kernicterus: Are these still problems in the third millennium? Neonatology. 2011;100: 354–62. doi: 10.1159/000330055 21968213 10.1159/000330055

[7] Johnson L, Bhutani VK, Karp K, Sivieri EM, Shapiro SM. Clinical report from the pilot USA Kernicterus Registry (1992 to 2004). J Perinatol. 2009;29: 25–45. 10.1038/jp.2008.211 19177057

[8] American Academy of Pediatrics Subcommittee on Hyperbilirubinemia. Management of hyperbilirubinemia in the newborn infant 35 or more weeks of gestation. Pediatrics. 2004;114: 297–316. 15231951 10.1542/peds.114.1.297

[9] Sgro M, Kandasamy S, Shah V, Ofner M, Campbell D. Severe neonatal hyperbilirubinemia decreased after the 2007 Canadian guidelines. J Pediatr. 2016;171: 43–7. doi: 10.1016/j.jpeds.2015.12.067 26852177 10.1016/j.jpeds.2015.12.067

[10] Demirel G, Akar M, Celik IH, Erdeve O, Uras N, Oguz SS, et al Single versus multiple dose intravenous immunoglobulin in combination with LED phototherapy in the treatment of ABO hemolytic disease in infants. Int J Hematol. 2011;93: 700–3. doi: 10.1007/s12185-011-0853-4 21617887 10.1007/s12185-011-0853-4

[11] Betran AP, Torloni MR, Zhang J, Ye J, Mikolajczyk R, Deneux-Tharaux C, et al What is the optimal rate of caesarean section at population level? A systematic review of ecologic studies. Reprod Health. 2015;12: 57 doi: 10.1186/s12978-015-0043-6 26093498 10.1186/s12978-015-0043-6 PMC4496821

[12] Lee BK, Le Ray I, Sun JY, Wikman A, Reilly M, Johansson S. Haemolytic and nonhaemolytic neonatal jaundice have different risk factor profiles. Acta Paediatr. 2016;105: 1444–50. doi: 10.1111/apa.13470 27173507 10.1111/apa.13470

[13] Battersby C, Michaelides S, Upton M, Rennie JM, on behalf of the Jaundice Working Group of the Atain. Term admissions to neonatal units in England: A role for transitional care? A retrospective cohort study. BMJ Open. 2017;7: e016050 doi: 10.1136/bmjopen-2017-016050 28554938 10.1136/bmjopen-2017-016050 PMC5726072

[14] Murki S, Kumar P, Majumdar S, Marwaha N, Narang A. Risk factors for kernicterus in term babies with non-hemolytic jaundice. Indian Pediatr. 2001;38: 757–62. 11463962

[15] Slusher TM, Zipursky A, Bhutani VK. A global need for affordable neonatal jaundice technologies. Semin Perinatol. 2011;35: 185–91. doi: 10.1053/j.semperi.2011.02.014 21641493 10.1053/j.semperi.2011.02.014

[16] Liu S, Wen SW, McMillan D, Trouton K, Fowler D, McCourt C. Increased neonatal readmission rate associated with decreased length of hospital stay at birth in Canada. Can J Public Health. 2000;91: 46–50. 10765585 10.1007/BF03404253 PMC6980127

[17] Maisels MJ. Screening and early postnatal management strategies to prevent hazardous hyperbilirubinemia in newborns of 35 or more weeks of gestation. Seminars Fetal Neonatal Med. 2010;15: 129–35. 10.1016/j.siny.2009.10.004 20034861

[18] Maisels MJ. Managing the jaundiced newborn: A persistent challenge. CMAJ. 2015;187: 335–43. doi: 10.1503/cmaj.122117 25384650 10.1503/cmaj.122117 PMC4361106

[19] Bhutani VK, Zipursky A, Blencow H, Khanna R, Sgro M, Ebbesen F, et al Neonatal hyperbilirubinemia and rhesus disease of the newborn: Incidence and impairment estimates for 2010 at regional and global levels. Pediatr Res. 2013;74(Suppl 1): 86–100. 24366465 10.1038/pr.2013.208 PMC3873706

[20] Altuntas N, Yenicesu I, Himmetoglu O, Kulali F, Kazanci E, Unal S, et al The risk assessment study for hemolytic disease of the fetus and newborn in a university hospital in Turkey. Transfus Apher Sci. 2013;48(3): 377–80. doi: 10.1016/j.transci.2013.04.021 23619329 10.1016/j.transci.2013.04.021

[21] Celik HT, Gunbey C, Unal S, Gumruk F, Yurdakok M. Glucose-6-phosphate dehydrogenase deficiency in neonatal hyperbilirubinemia: Hacettepe experience. J Paediatr Child Health. 2013(49): 399–402. 10.1111/jpc.12193 23573906

[22] Prior E, Santhakumaran S, Gale C, Philipps LH, Modi N, Hyde MJ. Breastfeeding after cesarean delivery: A systematic review and meta-analysis of world literature. Am J Clin Nutr. 2012;95: 1113–35. doi: 10.3945/ajcn.111.030254 22456657 10.3945/ajcn.111.030254

[23] Barber EL, Lundsberg LS, Belanger K, Pettker CM, Funai EF, Illuzzi JL. Indications contributing to the increasing cesarean delivery rate. Obstet Gynecol. 2011;118: 29–38. doi: 10.1097/AOG.0b013e31821e5f65 21646928 10.1097/AOG.0b013e31821e5f65 PMC3751192

[24] Danielson B, Castles AG, Damberg CL, Gould JB. Newborn discharge timing and readmission, 1992–1995. Pediatrics. 2000;106: 31–9. 10878146 10.1542/peds.106.1.31

[25] Bayoumi YA, Bassiouny YA, Hassan AA, Gouda HM, Zaki SS, Abdelrazek AA. Is there a difference in the maternal and neonatal outcomes between patients discharged after 24 h versus 72 h following cesarean section? A prospective randomized observational study on 2998 patients. J Matern Fetal Neonatal Med. 2016;29: 1339–43. doi: 10.3109/14767058.2015.1048678 26037723 10.3109/14767058.2015.1048678

[26] Erdeve O, Atasay B, Arsan S. Hypernatremic dehydration in breastfed infants: Is caesarean section a risk? Ann Trop Paediatr. 2005;25: 147–8. doi: 10.1179/146532805X45773 15949206 10.1179/146532805X45773

[27] Kuzniewicz MW, Escobar GJ, Newman TB. Impact of universal bilirubin screening on severe hyperbilirubinemia and phototherapy use. Pediatrics. 2009;124: 1031–9. doi: 10.1542/peds.2008-2980 19786442 10.1542/peds.2008-2980 PMC2858633

[28] Bjerre JV, Petersen JR, Ebbesen F. Surveillance of extreme hyperbilirubinaemia in Denmark. A method to identify the newborn infants. Acta Paediatr. 2008;97: 1030–4. doi: 10.1111/j.1651-2227.2008.00879.x 18482165 10.1111/j.1651-2227.2008.00879.x

[29] Gotink MJ, Benders MJ, Lavrijsen SW, Rodrigues Pereira R, Hulzebos CV, Dijk PH. Severe neonatal hyperbilirubinemia in The Netherlands. Neonatology. 2013;104: 137–42. doi: 10.1159/000351274 23887661 10.1159/000351274

[30] Mah MP, Clark SL, Akhigbe E, Englebright J, Frye DK, Meyers JA, et al Reduction of severe hyperbilirubinemia after institution of predischarge bilirubin screening. Pediatrics. 2010;125: e1143–8. doi: 10.1542/peds.2009-1412 20368324 10.1542/peds.2009-1412

[31] Manning D, Todd P, Maxwell M, Jane Platt M. Prospective surveillance study of severe hyperbilirubinaemia in the newborn in the UK and Ireland. Arch Dis Child Fetal Neonatal Ed. 2007;92: F342–6. doi: 10.1136/adc.2006.105361 17074786 10.1136/adc.2006.105361 PMC2675352

[32] Zoubir S, Mieth RA, Berrut S, Roth-Kleiner M, Swiss Paediatric Surveillance Unit. Incidence of severe hyperbilirubinaemia in Switzerland: A nationwide population-based prospective study. Arch Dis Child Fetal Neonatal Ed. 2011;96: F310–1. 10.1136/adc.2010.197616 21278431

[33] Mreihil K, Nakstad B, Stensvold HJ, Benth JŠ, Hansen TWR; Norwegian NICU Phototherapy Study Group; and the Norwegian Neonatal Network. Uniform national guidelines do not prevent wide variations in the clinical application of phototherapy for neonatal jaundice. Acta Paediatr. 2017 11 8. 10.1111/apa.14142 29119594

[34] Kumar P, Chawla D, Deorari A. Light-emitting diode phototherapy for unconjugated hyperbilirubinaemia in infants. Cochrane Database Syst Rev. 2011;7;(12): CD007969. 10.1002/14651858.CD007969.pub2 PMC6885069 22161417

[35] Mreihil K, Benth JŠ, Stensvold HJ, Nakstad B, Hansen TWR; Norwegian NICU Phototherapy Study Group; and the Norwegian Neonatal Network. Phototherapy is commonly used for neonatal jaundice but greater control is needed to avoid toxicity in the most vulnerable infants. Acta Paediatr. 2017 11 8 doi: 10.1111/apa.14141 29119603 10.1111/apa.14141

[36] Murki S, Kumar P. Blood exchange transfusion for infants with severe neonatal hyperbilirubinemia. Semin Perinatol. 2011;35: 175–84. doi: 10.1053/j.semperi.2011.02.013 21641492 10.1053/j.semperi.2011.02.013

[37] Hakan N, Zenciroglu A, Aydin M, Okumus N, Dursun A, Dilli D. Exchange transfusion for neonatal hyperbilirubinemia: An 8-year single center experience at a tertiary neonatal intensive care unit in Turkey. J Matern Fetal Neonatal Med. 2015;28: 1537–41. doi: 10.3109/14767058.2014.960832 25182682 10.3109/14767058.2014.960832

[38] Zipursky A, Bhutani VK. Impact of Rhesus disease on the global problem of bilirubin-induced neurological dysfunction. Semin Fetal Neonatal Med. 2015;20: 2–5. doi: 10.1016/j.siny.2014.12.001 25582277 10.1016/j.siny.2014.12.001

[39] Bhutani VK, Johnson L, Sivieri EM. Predictive ability of a predischarge hour-specific bilirubin measurement for subsequent significant hyperbilirubinemia in healthy term and near-tem newborns. Pediatrics. 1999;103: 6–14. 9917432 10.1542/peds.103.1.6

